# A novel lumen-apposing metal stent with an anti-reflux valve for endoscopic ultrasound-guided drainage of pseudocysts and walled-off necrosis: A pilot study

**DOI:** 10.1371/journal.pone.0221812

**Published:** 2019-09-04

**Authors:** In Rae Cho, Moon Jae Chung, Jung Hyun Jo, Hee Seung Lee, Jeong Youp Park, Seungmin Bang, Seung Woo Park, Si Young Song

**Affiliations:** 1 Department of Internal Medicine, Catholic Kwandong University College of Medicine, Incheon, Korea; 2 Department of Internal Medicine, Yonsei University College of Medicine, Seoul, Korea; National Institutes of Health, UNITED STATES

## Abstract

**Background:**

Pancreatic pseudocysts (PC) and walled-off necrosis (WON) are common complications of severe pancreatitis. Endoscopic ultrasound (EUS)-guided drainage has replaced surgery as the standard treatment for PC/WON. We developed a novel lumen-apposing metal stent (LAMS) with an anti-reflux valve to prevent infectious complications caused by food reflux into the cyst cavity. This retrospective study investigated the efficacy and safety of EUS-guided drainage using this LAMS.

**Methods:**

We investigated and compared the treatment outcomes and complications rates between EUS-guided drainage using a novel LAMS (n = 10) versus plastic stents (n = 18) from December 2013 to October 2016. Technical success was defined as successful stent placement without immediate complications. Clinical success was defined as resolution of the PC/WON and disappearance of symptoms.

**Results:**

Among 10 patients in LAMS group, 4 patients had complicated PC and 6 patients had WON. In the plastic stent group, 15 and 3 patients had PC and WON, respectively. The median fluid collection size before treatment was 82.5 (interquartile range [IQR], 60.75–118.25) mm and 92.0 (IQR, 75.75–130.25) mm in the LAMS and plastic stent groups, respectively. There were no statistically significant differences in technical success rates (90% vs. 94.4%; p = 0.999), clinical success rates (80% vs. 77.8%; p = 0.999), and complication rates (20% vs. 27.8%; p = 0.999) between the two groups.

**Conclusions:**

Treatment outcomes of EUS-guided drainage using a novel LAMS were feasible despite the significantly high proportion of WON. The LAMS allowed acceptable treatment outcomes for EUS-guided drainage.

## Introduction

Severe acute pancreatitis and chronic pancreatitis result in local complications in the form of peripancreatic fluid collections (PFC). According to the revised Atlanta classification, local complications of pancreatitis are classified as acute PFC, pancreatic pseudocyst (PC), acute necrotic collection, and walled-off necrosis (WON) [1]. Among these, PC and WON are encapsulated fluid collections with or without solid necrotic components that usually occur after more than 4 weeks since the onset of pancreatitis. Most PC and WON cases remain asymptomatic or resolve spontaneously. However, treatment is required when symptoms and complications, such as pain, biliary obstruction, or infection occur [2].

Previously, surgical interventions, such as drainage and open necrosectomy, were the only modalities of treatment, but these invasive procedures are associated with high rates of complications and mortality [3, 4]. With recent technological advances, endoscopic ultrasound (EUS)-guided drainage has replaced surgical treatment and has become the standard treatment [5]. The endoscopic approach has shown favorable treatment outcomes for PC and WON [6]. Studies have reported that success rates of EUS-guided fluid collection drainage and endoscopic necrosectomy range from 84% to 94% and 68% to 91%, respectively [7–9]. In addition to success rates, EUS-guided treatment has benefits regarding mortality, cost, hospital stay, and quality of life [10, 11].

EUS-guided treatment of PC and WON consists of fistula tract formation between the lesion and stomach (cystogastrostomy) or duodenum (cystoduodenostomy), stent placement via the fistula tract, and transluminal drainage. Therefore, for successful treatment, it is important for stent patency to be maintained without obstruction or migration throughout the treatment period. To date, a plastic stent has been used for EUS-guided drainage procedures. However, it is susceptible to obstruction and migration, and multiple stents are required to maintain adequate tract size for treating WON with solid necrotic debris [12]. The practice of placing multiple plastic stents is a time-consuming and difficult procedure because collapse of the cyst cavity and change of the tangential axis may occur after deployment of the first stent [13]. In recent years, metal stents have been developed to overcome the limitations of plastic stent with small calibers. The fully covered self-expanding metal stent (FCSEMS) and specially designed lumen-apposing metal stent (LAMS) are examples of these stents [14]. LAMS is a recently introduced novel “barbell-shaped” stent with larger calibers that allow for longer patency, reduced rates of occlusion, and decreased probability of secondary infections. Further, owing to the presence of bilateral anchor flanges, LAMS has anti-migrating ability and ability to oppose the PFC wall to the gastric or duodenal wall [15, 16]. However, according to a recent study, there is not enough current evidence to support the superiority of metal stents for transmural drainage of PFC compared to plastic stent. Therefore, further investigations of novel devices are needed [17].

We developed a novel fully covered LAMS with an anti-migration flap and anti-reflux valve to prevent complications such as stent migration and reflux of bowel contents, thereby improving stent patency and minimizing the fasting period after the procedure.

## Materials and methods

### Patients

We retrospectively reviewed patients who underwent EUS-guided drainage (cystogastrostomy or cystoduodenostomy) using a novel LAMS to treat PC or WON at Severance Hospital from December 2013 to October 2016. This study was approved by the local Institutional Review Board (4-2019-0114) and was conducted in accordance with the principles set forth in the Declaration of Helsinki. Given its retrospective nature, written informed consent was not required by the board to access the clinical data. Indications for EUS-guided drainage were infected PC or WON, gastric outlet obstruction, biliary obstruction, and intractable symptoms such as abdominal pain. The size of the PC or WON itself was not an indication for the procedure During this period, 10 and 18 patients underwent EUS-guided drainage using the novel LAMS (LAMS group) and conventional plastic stent (PS group), respectively. All pertinent data including demographics, lesion site, symptom improvement, duration of stent placement, and treatment-related adverse events were investigated. Technical success was defined as successful stent placement without immediate adverse events. Clinical success was defined as resolution of the PC/WON and the disappearance of symptoms.

### Materials

The novel HANARO stent (M.I.Tech, Seoul, South Korea) used in this study is a specially designed LAMS. This novel LAMS was designed to reduce side effects and improve treatment results of EUS-guided drainage.

**Fig 1:**
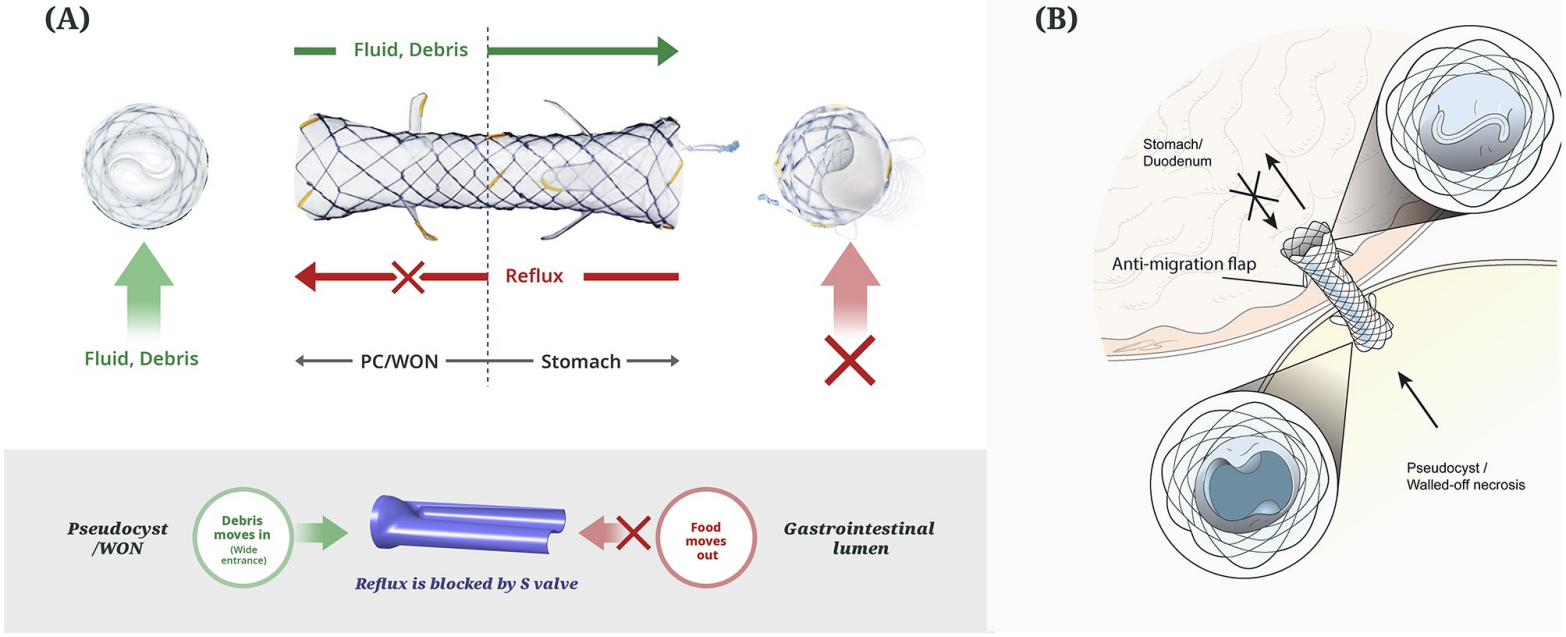
The lumen-apposing metal stent with an anti-reflux S-valve and anti-migration flaps (A) and schematic illustration of the anti-reflux S-valve inside the lumen of the lumen-apposing metal stent (B). This S-valve prevents the reflux of food from the gastrointestinal tract to the pancreatic pseudocyst/walled-off necrosis.

**Fig 2:**
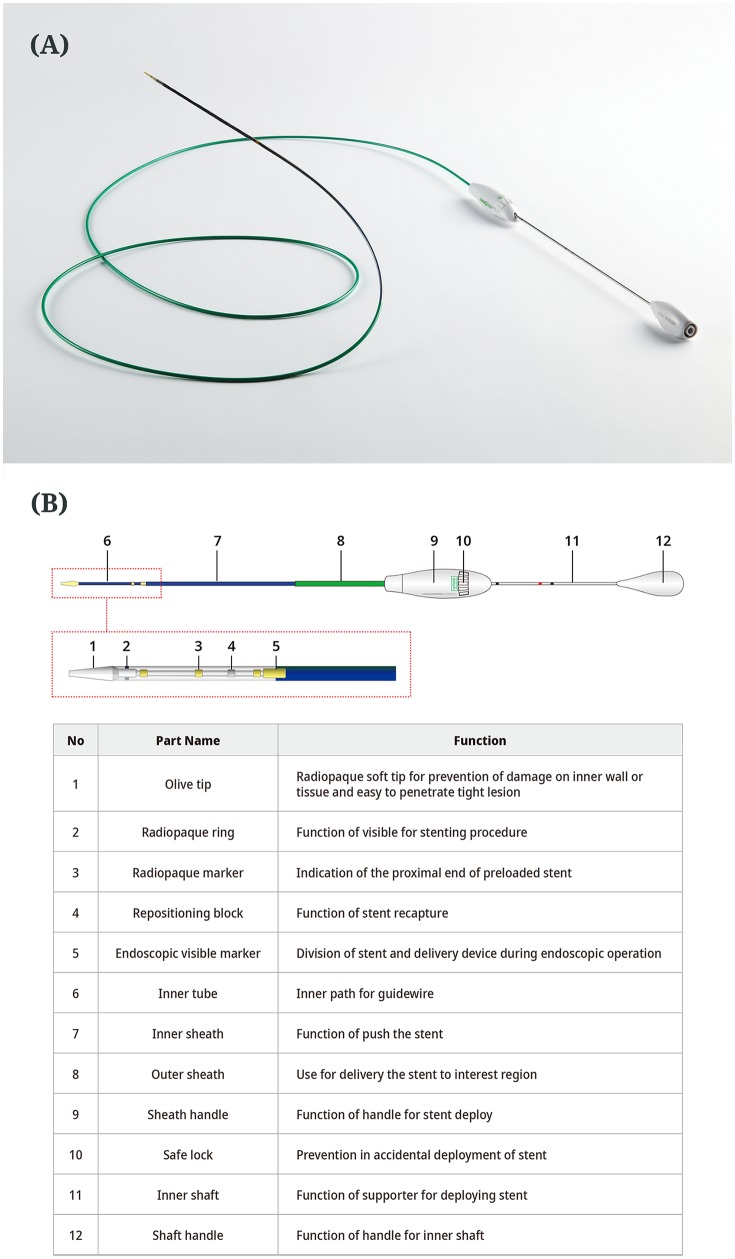
The 10.2 Fr delivery system for the novel lumen-apposing metal stent. Its gross appearance (A) and name and function of each part (B).

The LAMS has a structure of weaving nitinol mesh with full silicone covering. Stent dimensions of 10 mm diameter and length of 40 mm and 50 mm, were applied in accordance with the environment between the gastrointestinal lumen and PFC. For anti-migration, both ends of the stent are flared and additional 3 flaps are located on both sides. The S-type valve is applied to the internal path of the stent for anti-reflux. The function of the S-type valve is to drain the fluid and debris in the PFC into the gastrointestinal tract and prevent food reflux. The proximal end of the stent has a retrieval lasso for smooth stent removal from body (Fig 1A and 1B). The delivery device of 10.2 Fr uses a braided catheter to maximize flexibility and prevent kinking. Although anti-migration flaps and valves were added to this novel LAMS, conventional 10.2 Fr could be used to deploy the stent instead of a thicker delivery device. Therefore, additional friction was not required to pass through the accessory channel of the endoscope (Fig 2A and 2B).

The stent and delivery system used in this study was approved by the Korean government (Ministry of Food and Drug Safety, Approval No. 04–1242) and commercialized and sterilized using a standardized manufacturing process.

### Procedure

**Fig 3:**
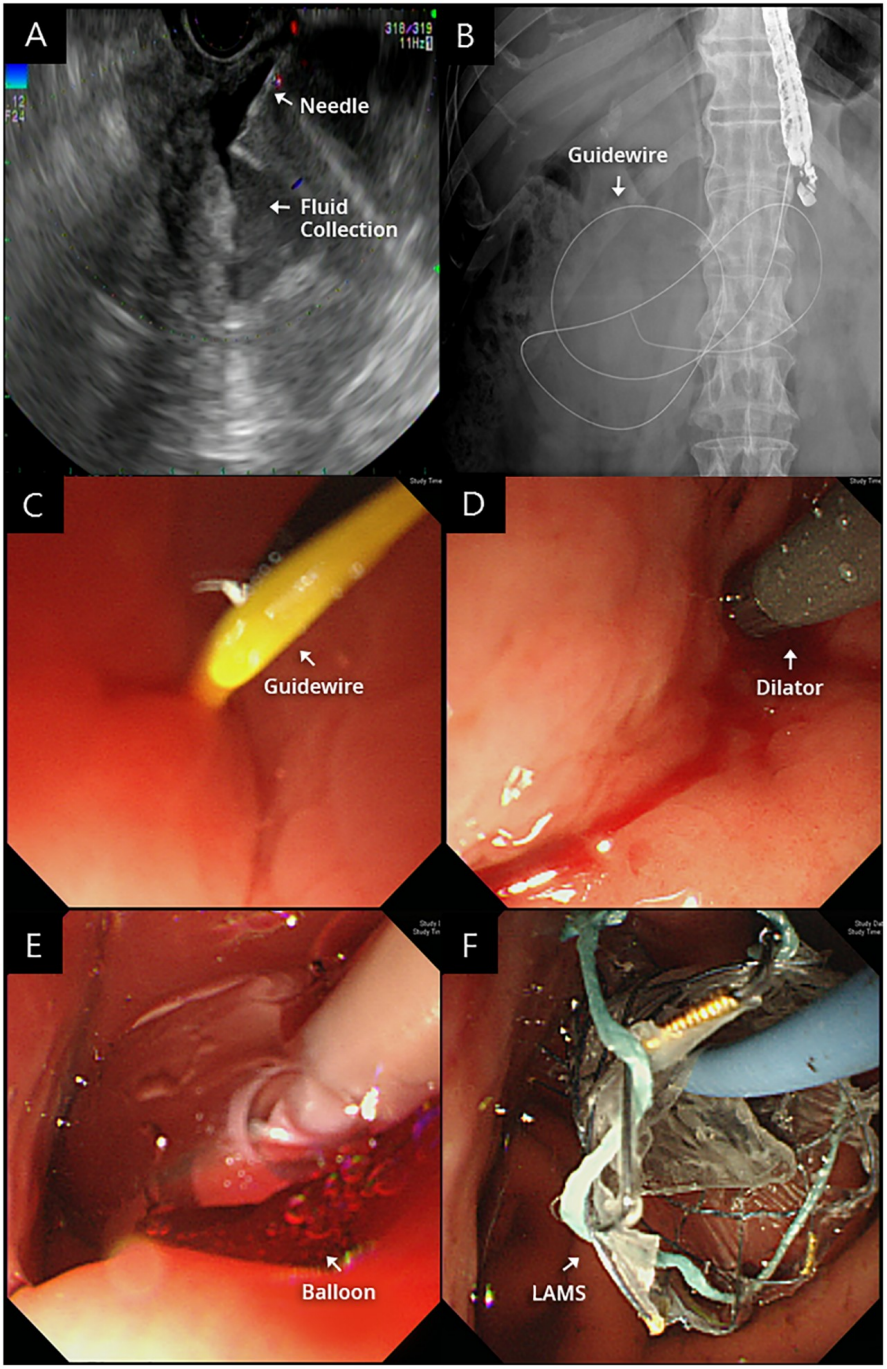
Endoscopic ultrasound (EUS)-guided pseudocyst drainage using the lumen-apposing metal stent (LAMS). (A) The lesion was punctured under EUS (Doppler) guidance with a 19-gauge EchoTip needle (B). (C) A guidewire was inserted in the lesion. (D) The puncture site was dilated using a Soehendra dilator. (E) Balloon dilatation using a Hurricane balloon. (F) The LAMS was deployed through the scope.

All procedures were performed by skilled endoscopists (M.J.C., J.Y.P., S.B., and S.W.P.). Patients were administered conscious sedation with midazolam and in case of poor sedation with midazolam alone, deep sedation was performed using propofol under cardiopulmonary monitoring. EUS GF-UCT260 (Olympus, Tokyo, Japan) scopes were used in all cases. Color Doppler imaging was used to identify interposed vessels, and each lesion was punctured under EUS guidance with a 19-guage EchoTip needle (Cook Medical, Bloomington, IN, USA). After the puncture, a straight VisiGlide (Olympus, Tokyo, Japan) guidewire was inserted into the lesion. The puncture site was dilated using a Soehendra dilator (Cook Medical), followed by balloon dilatation using a Hurricane balloon (Boston Scientific, Marlborough, MA, USA) if needed. Finally, the HANARO stent (M.I.Tech) was deployed via 10.2Fr delivery system through the scope (Fig 3). The LAMS was removed endoscopically after resolution of PC and WON using follow-up computed tomography approximately 6 to 8 weeks after the procedure.

### Statistical analysis

All statistical analyses were performed using SPSS for Windows version 23.0 (IBM, Armonk, NY, USA). Categorical variables were described as patient number and proportions for all patients. Continuous variables were described as medians and interquartile ranges.

## Results

### Treatment outcome of LAMS

The demographics, etiology, treatment details, and outcomes of all patients in LAMS group are summerized in Table 1. Ten patients (4 with PC and 6 with WON) underwent EUS-guided treatment using LAMS. The median size of fluid collection before treatment was 82.5 mm (interquartile range [IQR], 60.75–118.25 mm), and the median duration of stent placement was 47 days (IQR, 25.75–79.50 days).

**Table 1:** Data of all patients treated with LAMS.

	Age/Sex	Etiology	Reason for stent placement	Lesion size at diagnosis (mm)	Duration of stent placement (days)	Lesion size at follow-up (mm)	Technical success	Clinical success	Adverse event	Duration of NPO (days)	Additional treatment	Hospital days after stent placement	Procedure time (min.)
1	33/F	PC	Symptom	61	42	0	Yes	Yes	No	2	No	14	12
2	35/M	WON	Infection	64	30	0	Yes	Yes	No	3	No	11	29
3	43/M	WON	Symptom	92	355	22	Yes	Yes	No	1	No	23	29
4	59/M	PC	Symptom	143	71	21	Yes	Yes	Fever	1	No	12	36
5	58/M	PC	Infection	48	1	23	No	No	Peritonitis	11	Clipping & Nasogastric tube insertion	27	14
6	69/M	WON	Infection	214	13	220	Yes	No	No	1	Necrosectomy	108	65
7	42/M	PC	Gastric outlet obstruction	110	90	0	Yes	Yes	No	1	No	4	25
8	55/M	WON	Symptom	60	40	0	Yes	Yes	No	1	No	3	22
9	55/M	WON	Symptom	73	52	0	Yes	Yes	No	1	No	8	32
10	50/M	WON	Symptom	93	76	28	Yes	Yes	No	8	No	29	43

LAMS were placed successfully in 9 of 10 patients (unsuccessful placement in patient 5; 90% technical success rate). Clinical success was achieved by 8 patients (80%). One patient who did not achieve clinical success (patient #6) needed additional endoscopic necrosectomy after removal of the initially deployed LAMS. Adverse events were observed in only 2 patients. One patient experienced peritonitis due to immediate migration (patient #5). In this patient, LAMS was immediately removed and the gastric mucosa was sealed by endoscopic clipping. The patient recovered completely after non-surgical conservative care that included fasting, nasogastric tube insertion, and administration of broad-spectrum antibiotics. The other patient (patient #4) had fever for 3 days after the procedure but it improved after using antibiotics.

For most patients, oral feeding was resumed within 2 days after the procedure. Only 2 patients required more than a 1-week fasting period: 1 patient with an immediate complication and 1 with severe necrotizing pancreatitis. Patients who achieved clinical success did not require additional treatment to treat PC or WON. Removal of all LAMS was performed without adverse events.

### Comparison with plastic stent

Treatment outcomes and complications of EUS-guided drainage using LAMS were compared with those of EUS-guided drainage using plastic stent (n = 18) during the same period (Table 2). When patients were classified according to etiology, there was a significantly higher proportion of WON patients in the LAMS group than in the plastic stent group (60% vs. 16.7%; p = 0.035). There were no statistically significant differences in age, sex, or median size of fluid collection before treatment (82.5 mm vs. 92.0 mm; p = 0.524). There were no statistically significant differences in the median duration of stent placement (47 days vs. 55 days; p = 0.621), technical success rates (90% versus 94.4%; p = 0.999), clinical success rates (80% vs. 77.8%; p = 0.999), and adverse event rates (20% vs. 27.8%; p = 0.999). There was a trend toward shorter procedure times for the LAMS group than for the PS group (29 minutes vs. 37 minutes; p = 0.051), whereas the fasting periods after treatment (2.0 days vs. 2.0 days; p = 0.689) were not significantly different between both groups. In the plastic stent group, acute peritonitis developed in 2 patients, gastrointestinal bleeding developed in 1 patient, and stent self-migration (after resolution of fluid collection) developed in 2 patients.

**Table 2:** Comparison of treatment data between LAMS and plastic stent.

	Treated with LAMS (n = 10)	Treated with plastic stents (n = 18)	p-value
Age, years	52.50 (40.25–58.25)	48.00 (41.50–62.00)	0.981
Sex			0.375
Male	9 (90%)	13 (72.2%)	
Female	1 (10%)	5 (27.8%)	
Etiology			**0.035**
PC	4 (40%)	15 (83.3%)	
WON	6 (60%)	3 (16.7%)	
Lesion size at diagnosis, mm	82.50 (60.75–118.25)	92.00 (75.75–130.25)	0.524
Duration of stent placement, days	47 (25.75–79.50)	55 (30.75–101.50)	0.621
Technical success, n(%)	9 (90.0%)	17 (94.4%)	0.999
Clinical success, n(%)	8 (80.0%)	14 (77.8%)	0.999
Complication, n(%)	2 (20.0%)	5 (27.8%)	0.999
NPO duration, Days	2 (1–4.25)	2 (1–3)	0.689
HOD after treatment, Days	13 (7–27.5)	4.5 (4–11.25)	0.057
Procedure time, min	29 (20–37.75)	37 (33–48.5)	0.051

Variables are expressed as median (IQR) or n (%).

Abbreviation: NPO, Nulla per os (nothing by mouth); HOD, Hospital days

## Discussion

To date, double-pigtail plastic stent have been used for EUS-guided drainage for PC and WON. EUS-guided drainage using the plastic stent remains the standard treatment for PC because evidence indicating the superiority of metal stents for treating PC is not yet sufficient. The overall efficacy of the plastic stent for treating PC exceeds 90% [10, 18] the thin PC wall may be damaged by the tip of the metal stent owing to rapid collapse of the PC when it is treated with large-caliber metal stents. However, the efficacy of the plastic stent for WON treatment is significantly lower [19]. Because the plastic stent has a relatively small caliber (7 Fr or 10 Fr), the presence of solid debris in WON decreases drainage and increases the risk of secondary infection due to insufficient drainage of solid debris [19, 20].

Biliary or esophageal metal stents with large calibers were used to overcome the disadvantages of PS, but longer protrusions on both sides of the stents can cause contact ulceration in the bowel and late-phase bleeding [21]. There is also a risk of migration because the conventional straight esophageal and biliary metal stents have no anti-migration system such as flare ends and anti-migration flaps [22]. Moreover, although under-reported in the current literatures, there is a risk of stent obstruction or secondary infection due to retrograde reflux of food contents [23, 24]. Therefore, the specially designed LAMS was introduced for safer and more effective treatment of WON. Although controversies exist regarding the choice of stent for WON, recent studies have verified the superiority of the LAMS compared with the plastic stent for the treatment of WON. The use of LAMS was associated with fewer endoscopic sessions, fewer adverse events, shorter hospital stay, and a reduced need for salvage surgery [20, 25].

In this study population, both plastic stent and LAMS were used to treat PC/WON. When selecting a stent, LAMS was considered if patients had a large PFC requiring repeated drainage or showed walled-off necrosis in radiologic findings. In accordance with the previous studies, PC patients in the plastic stent group showed treatment success rate of 80% (12 in 15). However, 2 cases of stent migration and 3 cases of major adverse events (peritonitis, bleeding) were seen in the plastic stent group; however, stent migration after successful deployment was not observed in the LAMS group. Although this is a small pilot study, it is noteworthy that the newly designed LAMS did not differ in safety and efficacy when compared to plastic stent. The technical improvement applied to this LAMS prevented unwanted stent migration and cystic wall injury caused by metal stent tip, and there were no adverse events during the early resumption of oral feeding. Large scale studies are warranted to ensure that the technical improvements in stent structure and delivery system can minimize the chances of complications (i.e. migration, bleeding, etc.) and shorten the procedure time of EUS-guided drainage.

Previous clinical trials that verified the efficacy and safety of metal stents for PFC drainage are described by Ang et al [26]. When PFC was treated using a fully covered self-expandable metallic stent designed for drainage of PFC, technical success rates of 91% to 100%, clinical success rates of 76% to 100% were reported; however, the incidence of complications, such as bleeding (1% to 7%), perforation (1% to 2%), stent migration (1% to 6%), and infection (1% to 11%) was quite low [15, 16, 21, 22, 27–30]. Specially designed metal stents showed better outcomes than plastic stents in several studies, especially in the treatment of WON [20, 25, 31]. In our study using a novel LAMS, a high technical success rate (90%) and high clinical success rate (80%) were achieved, as in previous studies. The complication rate was also low; 1 case of fever and 1 case of immediate stent migration occurred. Therefore, this study outcome can support the clinical utility and safety of the use of LAMS in the treatment of WON and PC, which is specially designed for PFC drainage.

The presence of the anti-reflux valve inside the lumen and anti-migration flaps on each side are the distinguishing features of this novel LAMS. Several studies demonstrate the effect of preventing reflux when anti-reflux S-valve is applied to the metal stent [32–34]. Gastroesophageal reflux could be prevented by the esophageal metal stent with S-valve and longer duration of biliary metal stent could be achieved by preventing food reflux through the S-valve. Based on these studies, we conducted this pilot study and found a possibility of clinical improvement. In a previous study by Yamamoto et al. using a modified fully covered self-expanding metal stent, most patients fasted for a period of at least 3 days; however, in our study, most patients resumed oral feeding within 2 days, and there were no side effects related to food reflux [27]. A shorter duration of parenteral nutrition can reduce side effects and improve general patient conditions and compliance [35]. In addition, the presence of the anti-migration flap prevents stent migration and more accurately confirms the location and deployment of the distal flap on EUS or fluoroscopy during stent deployment.

This study had several limitations. First, it was a retrospective study with a small number of patients. Moreover, there was no precise planning for the long-term follow-up. Therefore, most patients were not evaluated after fluid collections resolved. As such, recurrence and late-phase complications might have been underestimated. Second, the patients were not randomly assigned to each group, and 10 patients cannot represent all the PC/WON patients. Thus, selection bias and sampling bias could have occurred. Further well-designed prospective studies are needed to validate these findings.

In conclusion, EUS-guided drainage using this novel LAMS demonstrated clinical utility to treat PFC with lower complication rates and acceptable treatment outcomes.
